# Oncogenesis as an Adverse Effect of Gene Replacement Therapy in Hematopoietic Stem Cells

**DOI:** 10.3390/ijms27146098

**Published:** 2026-07-08

**Authors:** Irina O. Petrova, Svetlana A. Smirnikhina

**Affiliations:** Laboratory of Genome Editing, Research Centre for Medical Genetics, Moskvorechye 1, 115478 Moscow, Russia

**Keywords:** oncogenesis, hematopoietic stem cells, gene therapy, viral vectors

## Abstract

Genetically modified hematopoietic stem cell therapy using gene-modified autologous hematopoietic stem cells has evolved over the last 30 years as an alternative approach to circumvent the limitations of donor availability, risks of excessive regimen related toxicity, prolonged immune suppression and graft-versus-host disease associated with allogeneic hematopoietic cell transplantation. Gene replacement therapy based on viral insertion of transgene into host genome was developed as one of the main methods for gene modification of autologous cells. Unfortunately, many cases of oncogenesis were directly caused by genetically modified hematopoietic stem cell therapy. The purpose of the present review is the description of cases of leukemogenesis in gene replacement therapy in hematopoietic stem cells, elucidation of the causes, and overview of the risk mitigation strategies. It aims to elucidate the main risk factors in gene replacement therapy in hematopoietic stem cells. The insertional mutagenesis leads to activation of proto-oncogenes, mostly *LMO2* and *MECOM-EVI1*. γ-retroviral vectors are dangerous in this case, as they contain long terminal repeats with strong promotor activity and are prone to integration near transcription initiation sites. Therefore, safer self-inactivating lentiviral vectors were developed, with long terminal repeats modified to reduce their promoter activity and with safer integration pattern. Nevertheless, the risk of leukemogenesis remains because the promoter integrated into the transgene expression cassette may still influence nearby gene expression. Another risk factor is monosomy 7, either pre-existing or caused by *MECOM-EVI1* activation, which may contribute directly to leukemogenesis. Thus, oncogenesis in HSPC gene replacement therapy does not have a single definitive cause; rather, multiple factors may contribute, and each may be sufficient under specific conditions.

## 1. Introduction

Gene replacement therapy is a prospective method for direct etiological treatment of genetic diseases. In the last decade, major progress was achieved particularly in ex vivo gene replacement therapy in hematopoietic progenitor stem cells (HPSCs) (Chancellor et al., 2023) [1]. HPSCs are specialized progenitor cells located primarily in the bone marrow that produce all blood cell types through a process called hematopoiesis. They are defined by their two key properties: self-renewal and multipotency. Allogeneic HPSC transplantation is a standard method for treatment of several metabolic disorders, in-born immunodeficiencies, lysosome storage disorders, and hemoglobinopathies, but it requires a suitable donor and poses a risk of graft-versus-host immune reaction. Graft-versus-host disease is a major contributor to the high cost and morbidity of allogeneic hematopoietic cell transplantation, which is also associated with 10–30% transplantation-related mortality due to relapse, organ failure, and infections (D’Souza et al., 2020) [2]. Therefore, the methods for genetic modification of autologous HPSCs are extremely desirable. One of the most prevalent variants of gene replacement therapy is based on the use of retroviral vectors (either γ-retroviral or lentiviral), which are able to insert the required transgene into the host genome (Petrova and Smirnikhina, 2025) [3]. Unfortunately, many cases of oncogenesis were directly caused by genetically modified hematopoietic stem cell therapy based on the viral vectors (Table 1). This fact is directly linked with the design of the gene therapy vectors. The main purpose of HSPC-based gene replacement therapy is to permanently alter gene expression in progenitor cells. This may create a lineage of cells with the potential for an abnormal proto-oncogene expression. This is a situation of a high oncogenic risk, especially as compared with cases of non-dividing cells or transient expression. The link between retroviral gene therapy and leukemia was a major historical setback for the field, but it also taught scientists invaluable lessons that have led to much safer therapies today.

As can be seen in Table 1, the risk of leukemogenesis remains an active concern in the currently available gene therapies. The design of new gene therapies must take into account the progress in the field to adhere to the best standards of safety available at the moment.

Note that Table 1 describes the cases of leukemia in approved and commercially available gene therapies. Table 2 presents an overview of cases of oncogenesis observed in clinical trials, including the trials for therapies from Table 1, but provides a more detailed picture. Note that the currently approved therapies are mostly based on lentiviral vectors, but the use of lentiviral vectors is a recent development, based on previous experience with γ-retroviral vectors.

There is an alternative method of genetic modification of HPSCs, namely, CRISPR/Cas genome editing. As compared to retroviral gene insertion, it preserves a normal gene copy number and upstream and downstream genomic context. Unfortunately, this method does not allow for insertion of a large sequence, but only for minor editing of the existing genome site (substitutions, insertions or deletions). In case of a complete loss of a gene copy or a major deletion, this feature prevents the use of CRISPR/Cas. It is preferably used to disrupt the functional genomic site, as in case of Casgevy, not to create a new function.

In this review, we aim to elucidate the risk factors for gene replacement therapy using retroviral vectors, some of which were successfully addressed, and some remain actual. The first part of review describes the viral vectors, the complicated history of their development, and the advances in their safety. The second part of the review describes the conditioning associated with cell therapy and its role in leukemogenesis.

## 2. γ-Retroviral and Lentiviral Vectors and Their Role in Oncogenesis

Early successful therapies used modified γ-retroviral vectors, such as Moloney murine leukemia virus (MMLV)-derived vectors, to deliver therapeutic transgenes. Integration of γ-retroviruses into the host genome is driven by the interaction of cellular BET family of proteins (BRD2, BRD3, and BRD4) with viral integrase. It should be noted, however, that insertional lymphomagenesis can occur independently from BET.

The primary reason certain early retroviral gene therapies caused leukemia is due to insertional mutagenesis, specifically through oncogene activation. Oncogenic potential of retroviruses is well-known. Oncogenic retroviruses can be divided into two groups, that is, acute transforming retroviruses and non-acute retroviruses (Maeda et al., 2008) [13]. Acute transforming retroviruses induce tumors due to expression of their viral oncogenes. Non-acute retroviruses induce tumors by activating cellular proto-oncogenes in the host cells; this results from insertion of proviral DNA in the vicinity of the activated proto-oncogene. The second mechanism creates the risk of oncogenesis for the retroviral gene therapy vectors. While most integrations are harmless, if the virus inserts itself in or near proto-oncogene areas, it can disrupt normal gene regulation and potentially cause oncogene overexpression. Vector insertions can contribute to transformation by the following mechanisms: (1) enhancer activation of proto-oncogene expression; (2) promoter insertion resulting in stimulation of expression from an immediately adjacent proto-oncogene; (3) insertional inactivation of a tumor suppressor gene; and (4) disruption of splicing or creation of 3′ mRNA truncations affecting stability, resulting in aberrant expression (Bushman, 2020) [14].

The first successful therapies used MMLV long terminal repeat (LTR) element as a promoter to express the transgene. The viral LTR is a strong promoter with well-known structure. The LTR is subdivided into three elements: U3, R, and U5 regions. The U3 region contains sequences necessary for the initiation of transcription, including basal promoter elements and upstream enhancers. Transcription of the provirus by cellular RNA polymerase II typically initiates at the U3–R boundary in the upstream LTR, and RNA cleavage/polyadenylation occurs at the R–U5 boundary in the downstream LTR (Maeda et al., 2008) [13]. When a provirus integrates upstream of a proto-oncogene in the same transcriptional orientation, the promoter and enhancer elements in one of the LTRs can increase the expression level of proto-oncogene by transcriptional read-through from the viral LTR, over-riding the normal transcriptional control of the proto-oncogene, a process termed promoter insertion (Maeda et al., 2008) [13]. As can be seen from the description of the cases found in the literature, promoter insertion was a major risk factor for leukemogenesis in γ-retroviral treatment.

The earliest and most well-documented cases of leukemia occurred in the early 2000s during trials for X-linked severe combined immunodeficiency (X-SCID). In X-SCID therapy, a defective Moloney murine leukemia virus carrying the *IL2RG* transgene was used to produce the vector, which was then used for transduction of autologous CD34+ cells (Cavazzana-Calvo et al., 2000) [15]. Clinical trials demonstrated several cases of T cell acute lymphoblastic leukemia (Hacein-Bey-Abina et al., 2010) [9]. Integration site analysis discovered that leukemogenesis was directly linked to insertions in the *LMO2* oncogene locus and in the *CCND2* locus. *CCND2* encodes cyclin D2, a known oncogene in lymphoid cells (Clappier et al., 2006) [16]. As of *LMO2*, it is a common insertion site for γ-retroviral integration due to the open state of chromatin in this locus in CD34+ cells, and its abnormal expression is strongly associated with T cell leukemia (Cattoglio et al., 2007) [17]. It is well accepted as a proto-oncogene for its specific capacity to transform normal thymocytes to leukemic cells due to ectopic expression (Wang et al., 2022) [18]. The fact that viral vectors included intact LTRs, which served as promoters and enhancers for *IL2RG* expression, was crucial for the activation of oncogenes. Similarly, insertion in the *LMO2* oncogenic site was the cause of leukemogenesis in ADA-SCID therapy, also based on MMLV vector (Cesana et al., 2024) [4].

In the case of chronic granulomatous disease, the SF71gp91pho retroviral vector was used for CD34+ transduction (Ott et al., 2006) [10]. In this vector, gp91phox expression is driven by the Friend mink cell Spleen focus-forming virus LTR, which has been shown to be highly active in stem and myeloid progenitor cells (Baum et al., 1995) [19]. Myelodysplasia with monosomy 7 was reported in several clinical trials, due to insertional mutagenesis in *MECOM, PRDM16* and *SETBP1* sites. *MDS-EVI1* complex locus (*MECOM*) is a well-known proto-oncogene involved in the genesis of myeloid leukemia and chromosomal instability (Stein et al., 2010a) [20]. Research by Stein et al. demonstrated that forced overexpression of *EVI1* in human cells can lead to disruption of normal centrosome duplication. This is a risk factor for genomic instability and may contribute to the loss of chromosome 7, a classic finding in therapy-related myelodysplasia that drives malignant progression (Breems et al., 2008) [21]. Monosomy 7 is linked to leukemogenesis, particularly in myeloid malignancies such as myelodysplastic syndrome and acute myeloid leukemia. It is one of the most well-established cytogenetic abnormalities associated with a poor prognosis. The loss of chromosome 7 leads to haploinsufficiency of key tumor suppressor genes located on that chromosome, such as *SAMD9*, *SAMD9L*, *EZH2*, and *MLL3* (Inaba et al., 2018) [22]. *EVI1* overexpression is associated with genomic instability, and the loss of chromosome 7 may further remove tumor suppressor genes, creating a highly aggressive, multi-hit leukemia.

In another study, a patient with chronic granulomatous disease was treated with the MMLV-derived MFGS-gp91^phox^ vector (Uchiyama et al., 2023) [11]. The patient developed myelodysplasia caused by vector integration into *MECOM* site 32 months after gene therapy. The initial anomaly in hematopoiesis, with possible secondary mutations, led to clonal dominance and subsequent development of leukemia. MFGS integration into *MECOM* locus yielded clonal expansion but did not induce chromosomal changes. The blast transformation occurred only after the acquisition of an additional genetic lesion—the biallelic loss of the *WT1* tumor suppressor gene—demonstrating a classic two-hit model of leukemogenesis. This result confirms that oncogenesis may have a complex origin and should not be reduced to the single risk factor. For example, the same 2023 case also revealed a novel mechanism: the *CYBB* transgene in myelodysplastic blasts contained more than 100 G-to-A mutations caused by APOBEC3 family enzymes. This hypermutation likely inactivated the therapeutic transgene, allowing the pre-malignant clone to evade any growth disadvantage and proliferate further. This is an important finding, as it elucidates the role of pre-existing genetic lesions in the development of leukemia, which is important to consider discussing the pre-treatment conditioning. The pre-treatment conditioning can have mutagenic effect (see the next section), thus amplifying the risk of malignant transformation.

Gene replacement therapy for Wiskott–Aldrich syndrome was developed on the basis of a gibbon ape leukemia virus (GALV)-γ-retroviral vector. In this vector, the MMLV LTRs are replaced with the corresponding myeloproliferative sarcoma virus LTRs and the normal MMLV tRNA primer binding site is replaced by a glutamine tRNA primer binding site (Klein et al., 2000) [23]. Later analysis demonstrated integration at the *LMO2*, *MECOM*, and *MN1* loci. MN1 is a transcriptional factor with a role in meningioma.

Because of the leukemogenic potential and technical limitations of γ-retroviral vectors, more recent gene therapy strategies have increasingly used lentiviral vectors, which are able to transduce non-dividing cells and are more flexible as vectors for gene replacement.

Unlike γ-retroviruses, the integration of lentiviruses is driven by (LEDGF)/p75 protein, which is a direct binding partner of lentiviral integrase. γ-Retroviral insertions cluster in regions near transcription start sites and associated features such as CpG islands and DNAseI hypersensitive sites (Wu et al., 2003) [24], and lentiviral insertions are favored throughout the entire gene, with both vector classes favoring sites in or near genes active in transduced cells (Bushman et al., 2005; Hematti et al., 2004) [25,26]. This distinct integration pattern contributes to a more favorable safety profile for lentiviral vectors and supports more reliable therapeutic outcomes when compared with γ-retroviral vectors. It should be noted that this does not mean that the integration profile of lentiviruses is fully safe. The integration inside the gene may lead to the insertional inactivation of a tumor suppressor or create aberrant splicing.

Patterns of integration for γ-retroviral and lentiviral vectors were analyzed for the case of CAR-T therapies (Guiraud et al., 2025) [27]. Peak expansion insertion site patterns were vector dependent: lentiviral CAR integrated mostly in introns and γ-retroviral vectors in intergenic regions, closer to transcription start sites, which is consistent with results for HPSCs (Montini et al., 2009) [28].

Self-inactivating (SIN) lentiviral vectors were developed for increased safety. SIN lentiviral vector is an engineered gene delivery vehicle, featuring a crucial deletion in the 3′ LTR U3 region. This modification, transferred to the 5′ LTR during reverse transcription, eliminates the LTR’s promoter/enhancer activity upon integration, reducing the risk of replication-competent retrovirus formation and mitigating insertional mutagenesis. The expression of transgene in this case is driven by an internal promoter. Thus, the probability of promoter activation of proto-oncogene upon integration of SIN vector is greatly decreased, as compared to vectors with unmodified LTRs. A potential transcriptional interference between the LTR and the internal promoter driving the transgene is also prevented by the SIN design.

**Figure 1:**
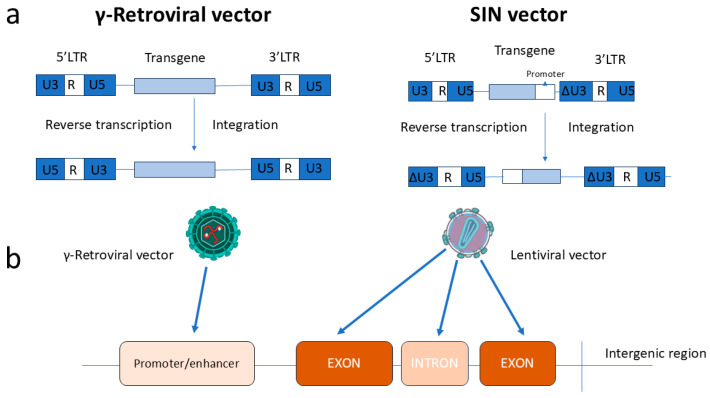
The progress in viral vector design is based on two factors. (**a**) The architecture of the first generation γ-retroviral and SIN vectors prior and after integration. Self-inactivating vectors include the deletion in the 3′ LTR region, which prevents the promoter activity of the viral LTRs. The addition of the internal promoter (often gene or cell lineage-specific) to the transgene expression cassette is required, making the design safer and more flexible. (**b**) Lentiviral vectors are used because, unlike γ-retroviral vectors, they integrate throughout the gene sequence and do not have a preference for promoter/enhancer regions.

Figure 1 summarizes the two factors that made progress possible in the viral vector design.

Unfortunately, the use of SIN lentiviral vectors does not fully prevent the leukemogenesis in gene therapy targeting CD34+ cells. The recent cases of hematological cancer were described in the patients who received lentiviral-based gene therapy for cerebral adrenoleukodystrophy (elivaldogene autotemcel) (Duncan et al., 2024) [5]. Lenti-D vector, which is used in elivaldogene autotemcel therapy, contains a virally derived synthetic regulatory element that includes the U3 segment of the myeloproliferative sarcoma virus long terminal repeat with the negative control region deleted and the DL587 endogenous retrovirus primer binding site substituted (modified MLV long terminal repeat promoter MNDU3). The MNDU3 promoter is derived from the U3 region of the myeloproliferative sarcoma virus (MND). It is a modified version of the viral LTR designed to act as a strong constitutive internal promoter. MNDU3 was chosen because it is a strong, ubiquitous promoter-enhancer that drives consistent gene expression across different cell lineages (Astrakhan et al., 2012) [29]. The overexpression of the transgene caused by the strong promoter was chosen to compensate for insufficient enzyme activity in the non-edited glial cells. Unfortunately, the strength of MNDU3 promoter is exactly the problem that creates the risk of leukemogenesis (Puig-Serra et al., 2025) [30]. The pattern of integration in that case was comparable with leukemia caused by γ-retroviral integration. The integration site analysis showed insertions in multiple loci, including *MECOM-EVI1*, *PRDM16*, *SMG6*, *SLCA16*, *INO80,* and more. The fraction of insertions into *MECOM* and its close homolog *PRDM16* was significantly higher in the patients involved in the described studies than in the patients involved in the other clinical studies of lentiviral vectors such as Zynteglo and Lyfgenia (Duncan et al., 2024) [5].

It should be noted that the oncogenesis in case of lentiviral-based gene therapy for cerebral adrenoleukodystrophy (elivaldogene autotemcel) (Duncan et al., 2024) [5] is design-specific and caused by the use of strong viral promoter MNDU3 in the transgene expression cassette. This promoter was chosen to ensure the elevated expression of the transgene. In other lentiviral vectors, the weaker, gene-specific promoters are used to achieve a physiological level of expression, thus preventing genotoxicity and activation of oncogenes.

Interestingly, acute myelogenous leukemia cells in a patient with sickle cell disease treated with HSPC lentiviral gene therapy contained a clonal insertion site near *VMA4*; however, this gene has never been implicated in cancer, and no change in expression of this or any other gene near the insertion site could be detected (Goyal et al., 2022; Kanter et al., 2023) [6,7]. The pre-existing monosomy 7 was the cause of leukemogenesis in this case. This case was deemed unlikely related to BB305 lentiviral insertion because of the insertion site location, low transgene expression in blast cells, and lack of effect on expression of surrounding genes. Genes of interest related to proto-oncogenesis (*MECOM* and *LMO2*) were not associated with the top 10 relative insertion site frequency in any patient. β-globin promoter, specific for erythroid cells, was used to ensure strictly regulated expression of anti-sickling β-globin in the vector in question. These findings suggest that, although insertional mutagenesis was observed, it did not play a major role in oncogenesis in this case.

The risk of oncogenesis can be reduced with the low viral vector copy number (VCN), which reduces the probability of insertional mutagenesis. The positive correlation between vector copy number and leukemogenesis was demonstrated experimentally in 2009 (Montini et al., 2009) [28]. A limit of two VCN is generally accepted to reduce the risk for insertional mutagenesis in hematopoietic stem cell-targeted gene therapy using integrating vectors; however, it might be insufficient for some disorders.

Some of the existing therapeutic vectors contain complex transgene expression cassettes, which include enhancer and/or control sites in addition to promoter. For example, the vectors for hemoglobinopathies (β-thalassemia and sickle cell disease) include locus control regions with long-range enhancer activity. These enhancers can potentially cause abnormal expression of genes in the vicinity of the provirus, but no cases of leukemogenesis associated with their activity were observed to date.

These findings provide evidence that the major risk factor for oncogenesis after viral-based gene replacement therapy is the proto-oncogene activation by strong viral promoters. In the case of γ-retroviral vectors, viral LTRs play the role of a promoter, so the oncogenic potential is intrinsic for these vectors. SIN lentiviral vectors were designed to remove the promoter activity of LTRs. Transgene expression, in this case, is driven by internal transgene promoter, so the choice of this promoter defines the level of transgene expression and the oncogenic potential of the vector. In case of leukemia linked to cerebral adrenoleukodystrophy treatment (Skysona), the strong retroviral promoter was used, and the result was similar to γ-retroviral vectors in regard to activation of proto-oncogenes. Thus, weaker and/or cell lineage-specific promoters are preferable to prevent oncogenesis by viral vectors.

## 3. Conditioning in Autologous HPSC Therapy

The analysis of clinical cases demonstrates that pre-existing genomic lesions, particularly the loss of the tumor suppressor genes, creates conditions for leukemogenesis under to the promoter insertion. Therefore, it would be helpful to discuss the isolation of target cells and the associated procedures.

The necessary step for ex vivo HPSC modification is the isolation of the cell population enriched with HPSCs. The target cells for gene modification are normally obtained by sorting based on the surface glycoproteins. CD34+ cells are the usual target for the HPSC gene modification. They are a population of cells found in bone marrow, cord blood, and peripheral blood, characterized by the expression of the CD34 surface glycoprotein. They are an enriched population containing stem and progenitor cells, although CD34 expression alone does not define true long-term hematopoietic stem cells. They are essential for reconstructing the blood and immune systems after myeloablative therapies for diseases like leukemia and lymphoma.

Ex vivo gene replacement therapy consists of several crucial steps, such as collection of the CD34+ cells from the peripheral blood or the bone marrow, gene modification with the use of the viral vectors, pre-transplantation conditioning, and transplantation of the modified cells. As can be seen from Table 2, not all cases of leukemia in gene replacement therapy are caused by insertional mutagenesis. Other risk factors include genotoxic conditioning, often performed prior to engraftment of modified CD34+ cells.

The goal of conditioning is the depletion of the host cells to create space for engraftment of genetically modified cells. Also, conditioning is necessary to provide the release of cytokines, which support engraftment as well (Fröbel et al., 2021) [31]. The immunosuppression, preventing the immune response to the transgene, is also a desirable goal. Therefore, the conditioning regimen has to be measured to achieve these goals without excessive toxicity.

The conditioning regimen often includes chemotherapeutic agents. These agents are administered prior to hematopoietic cell transplantation. They are characterized by DNA-alkylating activity or are nucleoside analogs. Typical examples include busulfan, cyclophosphamide, and fludarabine, which are associated with cytotoxicity due to the DNA damage (Bhattacharya et al., 2008) [32]. The lack of selectivity in the DNA damage caused by the conditioning factors creates a mutagenic environment, which can contribute to the emergence of pathogenic mutations.

The source of HPSCs plays a role in the choice of conditioning. Endogenous bone marrow ablation, stem cell support, and the extent of cytopenia are the considerations for choice of conditioning regimen (Bacigalupo et al., 2009) [33]. The regimens are classified by intensity as myeloablative, reduced intensity conditioning, and non-myeloablative.

Different diseases require different conditioning regimens. Disease-specific factors are a proportion of gene expression necessary, any requirement for organ specific expression, and any survival or engraftment advantage for gene-corrected populations. These factors affect the degree of cell depletion and/or immune suppression prior to engraftment which is necessary for phenotypic correction. For example, in the case of hemoglobinopathies, 30–50% of normal hemoglobin production is required, but full myeloablation has been necessary in gene-modified hematopoietic stem cell therapy for hemoglobinopathies to achieve sufficient engraftment of genetically modified cells (Okalova et al., 2025) [34]. Since the use of autologous cells theoretically eliminates the risk of graft rejection and graft-versus-host complications, stable chimerism of gene-modified and unmodified cells can be tolerated for some diseases. The important factor here is the extent of normal gene expression. This chimerism can be achieved using reduced-intensity regimens.

Myeloablative treatment with DNA-alkylating agents is required for patients with some metabolic conditions affecting nervous system in order to deplete resident immune cells in the central nervous system for new microglia, derived from genetically modified hematopoietic stem cells, to engraft (Tucci et al., 2021) [35]. Milder approaches are typically inadequate to achieve sufficiently high levels of engraftment of genetically modified hematopoietic stem cells required for phenotypical correction in case of hemoglobinopathies or lysosomal storage diseases. For metabolic diseases, due to the need for high enzyme expression levels as well as the risk of an immune response to the enzyme that is produced, fully myeloablative and immunoablative regimens are generally used to optimize outcomes. Immune ablation is beneficial for many of these disorders to reduce the risk of developing B cell or T cell mediated anti-enzyme immunity. The myeloablative process creates immense selective pressure. As the blood system regenerates from a limited number of engrafted, gene-corrected stem cells, any pre-malignant clone that has a growth advantage from the vector insertion can expand preferentially.

The insertional mutagenesis is not directly affected by conditioning, but the unmodified host cells suffer the mutagenic effect, the increased selective pressure which may cause clonal dominance, and the reduced immunity. All of these factors are oncogenic. For example, in Kanter et al. 2023 (Kanter et al., 2023) [7], one of the cases of leukemia was caused not by insertional mutagenesis, but by monosomy 7, and the authors attributed this to the use of busulfan. The monosomy itself may or may not be directly linked to the busulfan conditioning, as the leukemogenesis is a multifactorial process, as was stated earlier.

As can be seen in Table 1, the therapies for hemoglobinopathies (β-thalassemia and sickle cell disease), despite the use of SIN lentiviral vectors and lineage-specific promoters, present the risk of leukemogenesis. It could be linked to the specific conditions of the disease. The chronic inflammation, stress erythropoiesis, and bone marrow hypoxia associated with hemoglobinopathies may predispose the bone marrow to genomic instability, which can lead to higher rates of clonal hematopoiesis in this population, and the accumulation of somatic mutations. Pre-existing mutations can expand under the stress of genotoxic conditioning regimens, such as high-dose busulfan.

The role of conditioning in leukemogenesis is manifold. It contributes through the combination of several factors, such as direct mutagenesis due to the use of DNA-damaging agents, selective pressure favoring pre-existing clones, immune suppression, and marrow niche remodeling. Even when conditioning does not cause leukemogenesis directly, it may create a favorable medium for the leukemic clones created by viral insertion.

## 4. Conclusions

In conclusion, the higher incidence of leukemia in early HSPC trials was a tragic but crucial lesson in biology. It highlighted the immense power and risk of permanently modifying a stem cell with a poorly designed vector.

The retroviral gene replacement therapy caused leukemia primarily due to insertional activation of proto-oncogenes like *LMO2* or *MECOM* by powerful viral enhancers. Thanks to these insights, modern self-inactivating lentiviral vectors have dramatically improved the safety profile, making gene therapy a viable and life-saving treatment for many diseases today. According to the presently available data, lentiviral vectors pose a much smaller risk of leukemogenesis, because of their self-inactivating design. Self-inactivating vectors are characterized by the deletion in the U3 long terminal repeat region of the viral vector, which prevents the promoter activity of the vector backbone. The cases of leukemia observed after lentiviral gene modification were caused by the use of strong viral promoters as a part of transgene expression cassette, and were not directly connected to the lentiviral vector elements.

A particular risk is associated with metabolic diseases, where the requirement of the elevated transgene expression is combined with the necessary myeloablative conditioning. High expression requires stronger promoters with the risk of promoter insertion, and myeloablative conditioning causes the mutagenic environment accompanied by the reduced immunity. This combination of factors presents a high risk of leukemogenesis.

There are several possible ways to address the problem of oncogenesis in gene replacement therapy, which should be used in combination, namely: the use of lentiviral vectors; use of the self-inactivating vectors; weaker internal promoters; minimizing the vector copy number; screening for clonal hematopoiesis or cytogenetic abnormalities when appropriate; optimizing conditioning intensity; and performing long-term integration-site monitoring.

Nevertheless, the process of viral integration into the host genome and the subsequent insertional mutagenesis remains statistical. We cannot explain each particular case of oncogenesis or of lack of it without taking into account pure chance. But all these methods, which were developed for risk mitigation, were the product of searching for a balance between safety and efficiency. These methods are able to reduce the oncogenic risk to quite a significant extent, but post-treatment monitoring is still necessary and will be necessary in all cases.

## Figures and Tables

**Table 1:** The cases of oncogenesis in the ex vivo hematopoietic stem cell gene replacement therapies which currently entered the markets.

Commercial Name	Gene Delivery Method	Promoter	Disease	Oncogenesis
Strimvelis	replication- incompetent retroviral vector	Viral LTR	Adenosine deaminase deficiency	1 of 43 patients (Cesana et al., 2024) [4]
Skysona	lentiviral vector	MNDU3	Adrenoleukodystrophy	10 of 67 patients ((Duncan et al., 2024) [5]; https://www.hematologyadvisor.com/news/fda-restricts-skysona-use-after-more-reports-of-hematologic-malignancy/ (accessed on 27 June 2026))
Zynteglo	lentiviral vector	β-globin promoter	β-thalassemia	1 of 54 patients (Goyal et al., 2022) [6]
Lyfgenia	lentiviral vector	β-globin promoter	Sickle cell disease	2 of 9 patients (Kanter et al., 2023) [7]
Libmeldy, Lenmeldy	lentiviral vector	PGK promoter	Metachromatic leukodystrophy	No known cases
Waskyra	lentiviral vector	WAS promoter	Wiskott–Aldrich syndrome	No known cases
Kresladi	lentiviral vector	FES/CTSG human chimeric promoter	Leukocyte adhesion deficiency type I	No known cases

**Table 2:** The causes of oncogenesis in viral-based HPSC gene replacement therapy in hematopoietic stem cells.

Disease	Vector for Gene Therapy	Promoter	The Site of Oncogenic Insertional Mutagenesis	Causality	Reference
X-linked severe combined immunodeficiency	defective MMLV	Viral LTR	*LMO2, CCND2*	definite	Hacein-Bey-Abina et al. 2008, 2010 [8,9]
Chronic granulomatous disease	retroviral vector SF71gp91pho	Viral LTR	*MECOM, PRDM16, SETBP1*	definite	Ott et al. 2006 [10]
Chronic granulomatous disease	retroviral vector MFGS-gp91^phox^	Viral LTR	*MECOM*	definite	Uchiyama et al. 2023 [11]
Wiskott–Aldrich syndrome	GALV γ-retroviral vector	Viral LTR	*LMO2, MECOM, MN1*	definite	Braun et al. 2014 [12]
Adenosine deaminase deficiency	defective MMLV	Viral LTR	*LMO2*	definite	Cesana et al. 2024 [4]
Cerebral adrenoleukodystrophy	Lenti-D	MNDU3	*MECOM, PRDM16*	definite	Duncan et al. 2024 [5]
Sickle cell disease	BB305 lentiviral vector	β-globin promoter	No known oncogenes	unlikely	Kanter et al. [7]
β-thalassemia	BB305 lentiviral vector	β-globin promoter	No known oncogenes	unlikely	Goyal et al. 2022 [6]

## Data Availability

No new data were created or analyzed in this study. Data sharing is not applicable to this article.
